# Strength characterization of limestone lithofacies under different moisture states

**DOI:** 10.1038/s41598-025-22699-4

**Published:** 2025-10-21

**Authors:** Ahmed Gad, Hasan Arman, Saffet Yagiz, Osman Abdelghany, Bahaa M. Amin, Alaa Ahmed, Safwan Paramban, Mahmoud Abu Saima

**Affiliations:** 1 Geosciences Department, College of Science, United Arab Emirates University, Al Ain, 15551 United Arab Emirates; 2 School of Mining and Geosciences, Nazarbayev University, Astana city, 010000 Republic of Kazakhstan

**Keywords:** Carbonate rocks, Saturation conditions, Rock strength, Durability, Rus formation, Environmental sciences, Petrology, Civil engineering

## Abstract

This study characterized the mechanical behaviours of massive fractured (MLS), fossiliferous (FLS), and siliceous (SLS) limestone lithofacies under natural, dry, and saturated conditions. Uniaxial compressive strength (UCS), point load index (PLI), and indirect tensile strength (ITS) tests, along with petrographic, mineralogical, and geochemical analyses, were used to evaluate the impacts of the lithofacies composition and environmental conditions on rock strength. The results indicate that lithofacies composition, including mineralogy and texture, has a considerable effect on rock strength and durability. Under dry conditions, UCS values increased by up to ~ 200% in MLS and SLS relative to natural conditions, while saturation reduced UCS by 30–60% depending on lithofacies. Similar trends were observed in ITS, which decreased by up to 55% under saturation. The high silica content of SLS produced the most durable lithofacies, whereas the high porosity of FLS made it the most vulnerable to weakening from water exposure. MLS exhibited intermediate properties, as it loses strength considerably when existing fractures become saturated. Statistical analysis indicates that the CaO, SiO_2_, and MgO contents strongly influenced the rock mechanical properties. The study reveals relationships between lithofacies geochemistry, microstructural attributes (fractures, porosity, fossil interfaces), and mechanical responses under different moisture states. These insights allow for predictions about carbonate rock mechanical performance, making them crucial for geological research, engineering projects, industrial applications, and infrastructure design.

## Introduction

Limestone and dolostone are naturally heterogeneous carbonate rocks. These heterogeneities frequently result from complex interactions between the rock’s composition and its microstructures, which are typically inherited from their original deposition and subsequent diagenesis, as well as from saturating fluids with variable compositions. Understanding carbonate lithofacies is critical for sustainable resource use and determining effective engineering solutions^1–5^. Lithofacies represent the lithological and sedimentary features of sedimentary rocks, including their textures, mineralogical attributes, cementation characteristics, porosity, geochemical compositions, geometric relationships, and depositional environments, along with discontinuities (i.e. fractures and bedding planes). These sedimentary features inevitably have a considerable impact on the geotechnical behaviour of carbonate rocks^6–8^.

Climatic and environmental conditions such as saturation state, freeze–thaw cycles, and acid attack can extensively alter carbonate rock behaviour^9–12^. Saturated rocks are weaker than dry rocks and can be more easily deformed^13^. In addition, exposure to water can foster chemical weathering processes (i.e. karstification) that can lead to carbonate disintegration and deterioration, which are associated with geological disasters such as landslides^4,14^. In dry climates, particularly arid regions, rock expansion and contraction caused by daily temperature changes combined with periodic humidity, rain events, and thin soil cover may promote fracturing, which can enhance both surface and subsurface weathering^15,16^.

The geotechnical characteristics of carbonate rocks, such as strength, deformation style, porosity, density, and brittleness, are strongly and directly influenced by lithofacies type^5,7,17,18^. Rock strength is a critical geotechnical parameter that directly influences site selection, slope stability, and foundation design. In carbonate terrains, strength variability across lithofacies complicates engineering assessments and necessitates laboratory characterization of compressive, tensile, and shear properties^17–21^. Furthermore, these data are an important component of the predictive models that simulate rock behavior under diverse loading and environmental conditions. Recent advances in numerical and machine learning approaches (e.g. principal component regression, multivariate regression, least squares regression, gradient boosting machines, and least square support vector machine models) further enhance the accuracy of rock strength prediction, improving risk management in engineering design^22–26^.

Recently, researchers have attempted to determine the effects of saturation conditions on the physical and mechanical properties of carbonate rocks. Baechle et al.^27^ found that water saturation in carbonate rocks led to changes in their compressional and shear-wave velocities, as well as considerable changes in their shear moduli, with some samples exhibiting compressional velocities up to 500 m/s higher than those of dry samples. Vásárhelyi^28^ examined uniaxial compressive strength (UCS), tangent elastic modulus (*E*), and Brazilian tensile strength (BTS) data from 45 Miocene limestone blocks under dry and saturated conditions and determined that the saturated parameter values were 34% lower than those of the dry parameter values. Abd El Aal et al.^29^ found that saturation levels affect the strength and durability of limestone and marly limestone, with higher saturation levels decreasing the rock strength and durability and increasing the P-wave velocity. Habibi et al.^30^ observed substantial decreases in the tensile strengths of dolomite samples in the presence of distilled water and seawater. Compared with the dry sample, the seawater-saturated sample had a 16% lower tensile strength. Davarpanah et al.^31^ examined marl samples under air-dried, water-saturated, and frozen (-20 °C) conditions. They reported that the frozen samples had an average UCS of 21.93 MPa, which was 86.4% higher than that of the saturated (11.76 MPa) and 25.9% lower than that of the dry (29.62 MPa) specimens, respectively. Ajalloeian et al.^18^ investigated Cretaceous carbonate samples from Shahrekord, Iran, and found that their mechanical properties were influenced by dolomite percentage, grain size, allochem percentage, and carbonate percentage. Arman et al.^7^ conducted comprehensive in situ and laboratory Schmidt hardness value (SHV) analyses of carbonate-dominated rocks and found varying relationships between the mean SHV and UCS, which they attributed to the compositional and textural variability of the rocks, as well as to environmental variables that influence rock mechanical properties. Subsequently, Arman et al.^5^ found that iron oxide cement and silica content affect the mechanical properties of carbonate rocks. In addition, they demonstrated that the occurrence of dolomite and sulfate minerals may increase the rock strength.

Laboratory analyses of dry and/or saturated homogenous rock samples have frequently been used to investigate their physical and mechanical behaviours under different conditions. However, it is also critical to understand how changes in dryness and saturation conditions, as well as different carbonate lithofacies, affect rock mechanical properties. In this study, analyses of lithofacies-specific geochemical compositions and multi-condition mechanical testing (natural, dry, and saturated) were integrated to quantify the impacts of interactions between composition and environmental exposure on rock strength and durability. Previous studies have focused only on either individual lithofacies or saturation effects. Therefore, the goal of this study was to highlight the importance of investigating the impacts of both the lithofacies properties (i.e. mineralogy, texture, and geochemistry) and water saturation-dryness conditions on carbonate rock strength.

## Methods

### Geologic setting

**Fig. 1:**
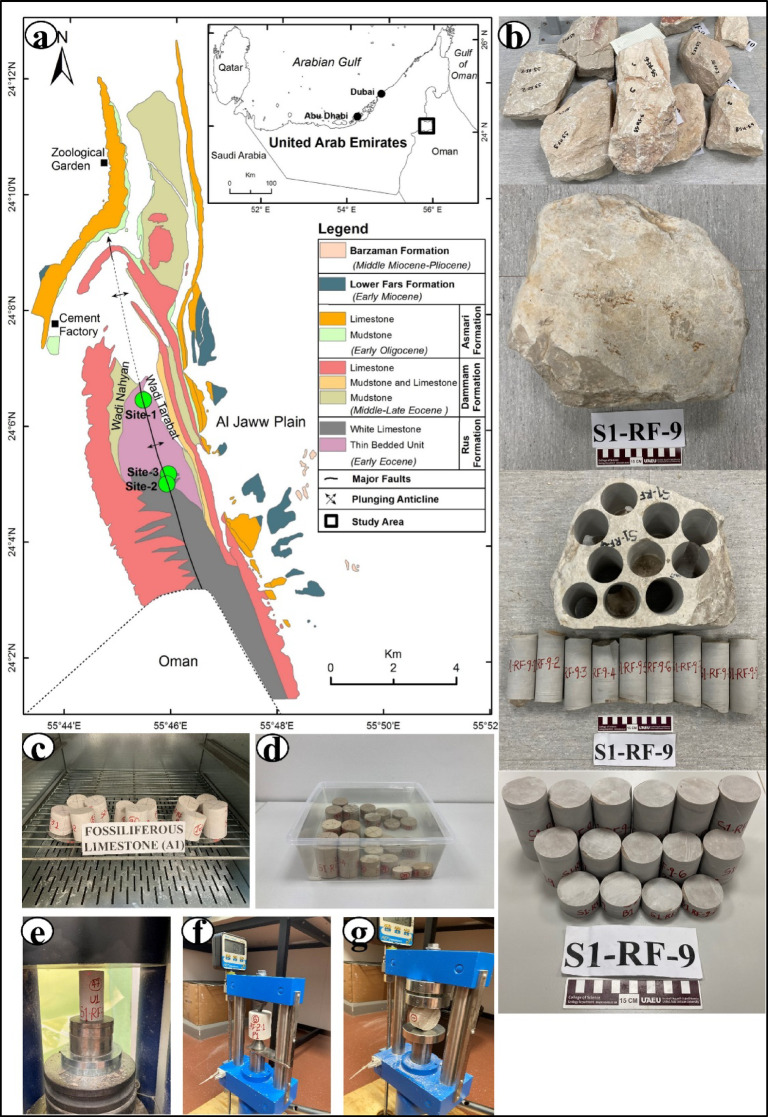
(**a**) Geologic map of the Hafit anticline showing sampling sites (generated with ArcGIS 10.8^35^). (**b**) Examples of rock, core, and test samples used for the analyses. Samples during the (**c**) drying and (**d**) saturation processes, as well as during the (**e**) UCS, (**f**) PLI, and (**g**) ITS analyses.

The carbonate rocks examined in this study belong to the early Eocene Rus Formation (55–49 Ma). This formation extends throughout Abu Dhabi and various parts of the Middle East, and is known for containing substantial oil reservoirs. The Rus Formation is exposed in the core of Hafit Mountain, south of Al Ain, where it reaches a stratigraphic thickness of 200 m (Fig. 1a). The upper Rus Formation comprises ~ 70 m of massive limestone, whereas the lower part of the formation comprises 130 m of nodular and thinly bedded marls and limestone. In the lower part of the formation, bands of irregularly shaped chert nodules less than 10 cm in diameter are mixed with fine-grained, muddy, and microfossil-rich limestones^32–34^.

### Sampling and sample Preparation

Thirty-seven representative rock samples were collected from an exposure of the Rus Formation located along the main road cut at Hafit Mountain (Fig. 1a) for geotechnical (i.e. mechanical and physical), textural, mineralogical, and geochemical analyses. The limestone samples were classified into three lithofacies according to the petrography of the fresh samples and their corresponding thin-sections: MLS (nine samples), FLS (13 samples), and SLS (15 samples).

Limestone blocks without macroflaws were transported to the laboratory, where NX-size (54 mm) cores were obtained. After coring, the samples were trimmed at both ends to ensure an appropriate core length and maintain a length-to-diameter ratio of approximately 2:1 for geotechnical analysis (Fig. 1b). Both ends of each sample were identical and within the defined measurement limits. Prior to analysis, the sample diameters and lengths were measured, and the samples were weighed. The air-dried samples were then ground into a fine powder using an agate mortar and stored in polyethylene bags until analysis.

### Analytical methods

The sample lithofacies (i.e. MLS, FLS, and SLS) were classified using a polarised light microscope (Nikon, LV 100 N POL) with the NIS Elements imaging software. Mineralogical compositions were determined using the X-ray diffraction (XRD) technique (Compact Aeris system equipped with an Empyrean X-ray tube Co LFF and a PIXcel1D line detector). To determine the major and trace elemental compositions, the X-ray fluorescence (XRF) analyses were performed on the powdered (< 74 μm) samples (Epsilon 1– EDXRF with SDD10P, MCA TYPE: PAN-DPP-Compact Detector and Ag50 Tube, Kv: 7.000–50.000, max. µA = 1000, max. Watts = 10.000).

Uniaxial Compression Strength (UCS), Point Load Index (PLI), and Indirect Tensile Strength (ITS) tests were performed according to the American Society for Testing and Materials (ASTM) standards^36–38^. All tests were performed under natural, dry, and saturated conditions. For natural conditions, samples were placed in an oven at room temperature (24 °C) for 12–16 h, to achieve equilibrium moisture content before coring, whereas the dry samples were placed in an oven at 105 °C for 12–16 h. Saturated conditions were obtained by saturating the samples with distilled water for 48–50 h (Fig. 1c–g).

To determine the percentages of non-carbonate components (e.g. chert, anhydrite, siliciclastic sand, silt, and argillaceous material), all samples were subjected to acid-insoluble residue analyses^39^.

All sample preparation, petrographic observations, and XRD, XRF, and rock strength analyses were conducted in the Geosciences Department of the College of Science, United Arab Emirates University. To ensure satisfactory XRD and XRF analytical performance, all operating parameters were software-controlled. The XRD and XRF analyses were also calibrated using standard reference materials (45W9202-Calcite, A1070-3590A5, and Cu-3590C2). All reagents used for the analysis were of analytical grade, and all solutions were prepared using double-distilled water.

## Results and discussion

### Petrography, Mineralogy, and geochemistry

**Fig. 2:**
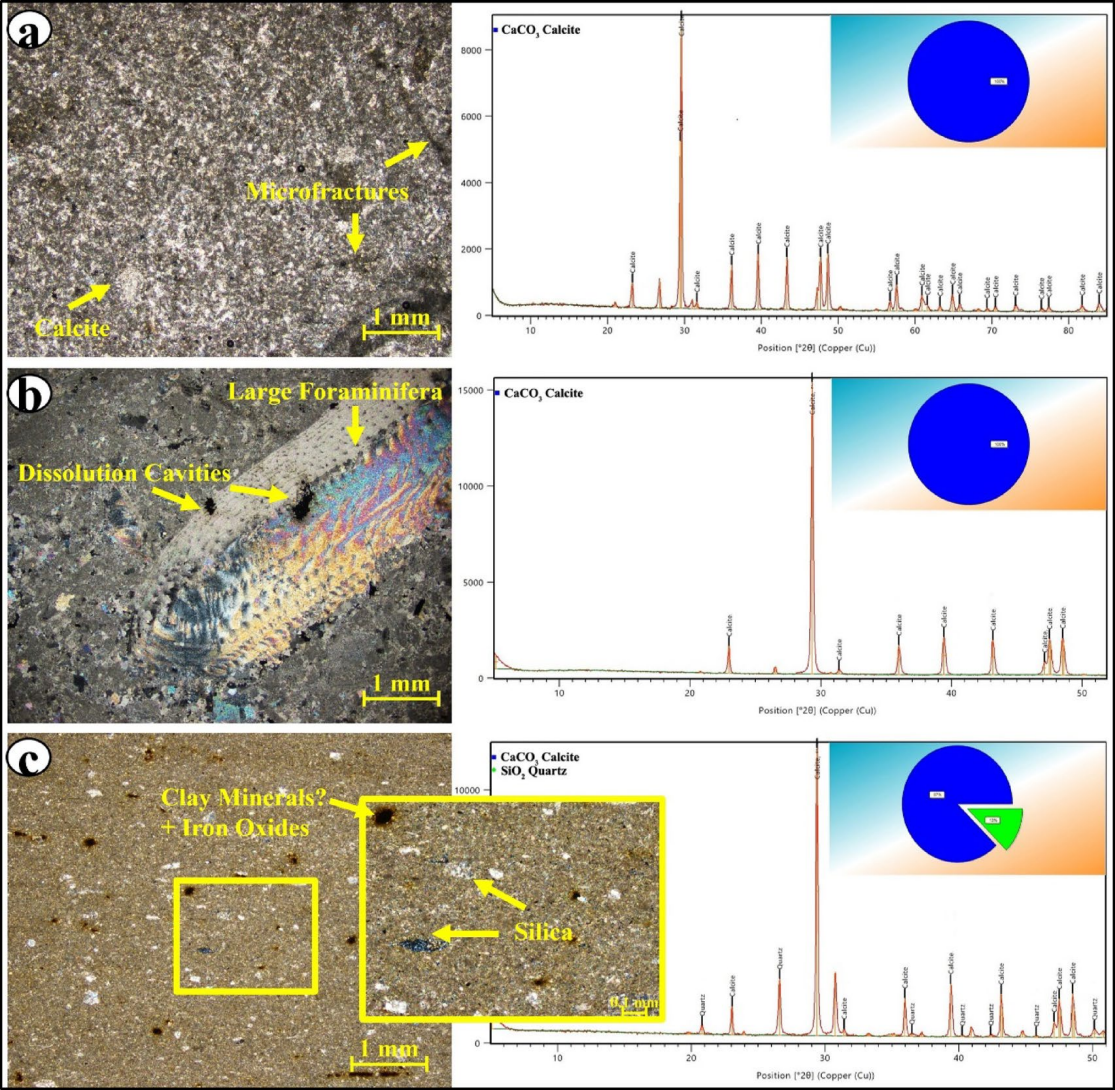
Cross-polarised light microscopy images and X-ray diffraction (XRD) patterns showing the texture and mineralogy of the (**a**) massive fractured (MLS), (**b**) fossiliferous (FLS), and (**c**) siliceous (SLS) limestone lithofacies.

Field observations and thin-section analyses of the Rus Formation exposed at Hafit Mountain identified three dominant lithofacies: massive fractured limestone (MLS), fossiliferous limestone (FLS), and siliceous limestone (SLS). MLS was characterised by a compact and uniform calcite crystalline texture (Fig. 2a). Its low porosity indicates limited secondary dissolution or alteration; however, the presence of fractures indicates that structural deformation occurred. The petrographic and thin-section analyses revealed that the fossiliferous limestone (FLS) lithofacies is rich in fossils, which are mainly large foraminifera and shell fragments (Fig. 2b). The remaining portion consists primarily of micritic to sparry calcite cement, which is similar in composition to the massive fractured limestone (MLS) but is texturally different due to the presence of fossil-matrix interfaces. The abundant fossils suggest a depositional environment with considerable biological activity (e.g. a shallow marine environment). SLS was characterised by a carbonate matrix mixed with micro-chert particles and clay minerals (Fig. 2c). The presence of silica and clay minerals indicates that SLS either received terrigenous inputs or experienced changes in depositional energy conditions^40–42^.

The X-ray diffraction (XRD) patterns of the lithofacies confirmed that the mineralogical compositions of the MLS and FLS consisted of carbonate minerals (calcite) (Fig. 2a, b). The absence of quartz and clay mineral peaks suggests that MLS and FLS have mostly carbonate compositions. The XRD pattern of SLS contained peaks corresponding to calcite and siliceous minerals (i.e. quartz or microcrystalline chert; Fig. 2c). Clay minerals were observed petrographically in SLS, but their proportions may fall below the XRD method’s detection limit for individual minerals, explaining the absence of distinct clay peaks in Fig. 2c. This is in line with the very low Al_2_O_3_ concentration measured in SLS by XRF (< 0.5 wt%; Table 1).

The minimum, maximum, and average major oxide contents of the limestone lithofacies are presented in Table 1. The X-ray fluorescence (XRF) analyses indicate that the three lithofacies have distinct variations indicative of differing depositional environments and diagenetic processes (Table 1). MLS samples had high CaO (95.6–98.8%) and low silica (0.32–2.37%) contents, with negligible amounts of Al_2_O_3_, Fe_2_O_3_, and MgO, which suggests minimal impurities^43^. This mineralogy is indicative of a stable low-energy marine environment with limited terrigenous inputs. The absence of fossils in MLS indicates that homogeneous sedimentation occurred. FLS samples had CaO contents that were slightly higher than those of MLS (97.6–99.1%), indicating a predominantly biogenic origin (e.g. fossils and shell fragments). The SiO_2_ contents were low (0.27–1.05%), while all other oxide contents were minimal. These characteristics indicate that FLS formed in a biologically active shallow marine zone, in which fossil debris contributed to the high CaO contents. SLS samples had lower CaO (78.5–96.4%) and higher SiO_2_ (2.17–14.04%) contents, thereby indicating that substantial silica input occurred. The relatively high Al_2_O_3_ (0.49%) and Fe_2_O_3_ contents of the SLS samples indicate clay or iron oxide impurities. Al_2_O_3_ is used as a proxy for clay content in limestone^44^; thus, the SLS lithofacies is shale-contaminated, as the average Al_2_O_3_ content of siliciclastic-contaminated carbonates is 0.42%^45^. The observed elevated MgO concentrations may indicate dolomitization, whereas the high silica contents and impurities indicate a combination of biogenic and terrigenous deposition processes or diagenetic silica enrichment. The MLS was very pure, as indicated by its low insoluble residue (IR) values (0.0–3.6), whereas SLS had the highest IR values (0.4–6.4), likely owing to terrigenous impurities (i.e. clay and silica).

The trace elemental compositions of the lithofacies (Table 1) indicate that they formed in distinct depositional environments. FLS had a similar elemental composition to that of MLS, but contained slightly elevated levels of Sr and Ag, which are likely indicative of biological processes such as skeletal aragonite formation or organic matter enrichment. SLS contained elevated Ti concentrations of up to 1400 ppm (average of 349 ppm), indicating that terrigenous inputs originating from clastic sediment occurred during its deposition. MLS and FLS contained low Zr concentrations and essentially zero Ti, thereby indicating a limited detrital input that occurred during deposition, in accordance with pure carbonate deposition in a stable marine setting. Elevated U (up to 50 ppm), V (up to 270 ppm), and Mo (up to 30 ppm) observed in the SLS samples, indicate that deposition occurred under suboxic to anoxic conditions, likely within an isolated basin. Low U contents (≤ 30 ppm) and low V/Mo ratios in the MLS and FLS samples suggest that these facies were deposited in oxic conditions typical of shallow marine environments with robust water circulation^46^.

**Table 1:** Major oxide and trace elemental compositions of the MLS, FLS, and SLS lithofacies.

Lithofacies		CaO	SiO_2_	MgO	Al_2_O_3_	Fe_2_O_3_	Na_2_O	*P*_2_O_5_	SO_3_	Cl	IR
MLS	Min.	95.59	0.32	BDL	BDL	0.04	BDL	BDL	0.02	0.04	0.00
	Max.	98.80	2.37	BDL	0.56	0.67	1.01	0.98	0.15	0.08	3.55
	Avg.	97.77	0.94	-	0.06	0.16	0.29	0.13	0.05	0.06	1.26
FLS	Min.	97.59	0.27	BDL	BDL	0.03	BDL	BDL	0.01	0.03	0.37
	Max.	99.14	1.05	BDL	BDL	0.09	0.59	0.09	0.28	0.08	6.42
	Avg.	98.80	0.45	-	-	0.05	0.08	0.01	0.05	0.05	2.82
SLS	Min.	78.52	2.17	BDL	BDL	0.11	BDL	BDL	0.05	0.04	1.85
	Max.	96.41	14.04	7.66	2.07	2.14	BDL	0.31	0.16	0.25	49.22
	Avg.	91.69	5.39	0.65	0.49	0.62	-	0.17	0.10	0.10	10.34

		Sc	Ti	V	Cr	Mn	Ni	Cu	Zn	Rb	Sr
MLS	Min.	BDL	BDL	80.0	40.0	30.0	30.0	BDL	BDL	BDL	280.0
	Max.	940.0	390.0	220.0	190.0	60.0	60.0	10.0	20.0	10.0	490.0
	Avg.	726.7	43.3	114.4	83.3	40.0	40.0	3.3	7.8	1.1	355.6
FLS	Min.	610.0	BDL	30.0	BDL	20.0	20.0	BDL	BDL	BDL	210.0
	Max.	950.0	BDL	170.0	80.0	40.0	40.0	10.0	10.0	BDL	540.0
	Avg.	773.1	BDL	97.7	40.0	29.2	29.2	0.8	3.8	BDL	352.3
SLS	Min.	BDL	BDL	50.0	80.0	20.0	20.0	BDL	10.0	BDL	300.0
	Max.	1620.0	1400.0	270.0	560.0	90.0	90.0	20.0	120.0	40.0	5110.0
	Avg.	458.0	349.3	103.3	156.7	45.3	45.3	5.3	34.7	10.7	1993.3

		Zr	Mo	Ag	Sn	Sb	Te	Cs	Ba	Er	U
MLS	Min.	BDL	BDL	2510.0	200.0	70.0	190.0	30.0	150.0	BDL	10.0
	Max.	10.0	10.0	4210.0	360.0	120.0	340.0	70.0	250.0	40.0	30.0
	Avg.	1.1	3.3	3095.6	258.9	87.8	238.9	45.6	191.1	5.6	22.2
FLS	Min.	BDL	BDL	2680.0	200.0	70.0	190.0	20.0	130.0	BDL	10.0
	Max.	10.0	BDL	3540.0	290.0	100.0	270.0	80.0	250.0	10.0	40.0
	Avg.	3.1	BDL	2956.2	245.4	83.1	227.7	46.9	186.9	0.8	18.5
SLS	Min.	BDL	BDL	2870.0	220.0	60.0	210.0	20.0	160.0	BDL	10.0
	Max.	50.0	30.0	4880.0	410.0	140.0	370.0	100.0	320.0	50.0	50.0
	Avg.	20.0	7.3	3224.7	265.3	90.0	246.7	49.3	230.7	24.0	25.3

BDL = Below detection limit.

### Rock strength

Table 2 summarises the physical and mechanical properties of the MLS, FLS, and SLS lithofacies. FLS exhibited the highest average density (*ρ*_*n*_, 2.6 g/cm³) and natural unit weight (*γ*_*n*_, 2.6 kN/m^3^). SLS had the highest Schmidt hardness value (SHV, 62), thereby demonstrating its high surface hardness. Although MLS produced lower average values, it exhibited uniform parameters. The SHV range for SLS (50–76; range = 26) is notably wider than those of MLS (48–66; range = 18) and FLS (49–67; range = 18). This broader spread in hardness values supports a higher degree of heterogeneity in SLS, which is consistent with the petrographic and XRF observation of variable silica and impurities content. These differences reflect the distinct compositional and textural characteristics of each lithofacies.

**Table 2:** Physical and mechanical properties of the MLS, FLS, and SLS lithofacies.

Lithofacies		ρ_n_ (gr/cm^3^)	γ_n_ (kN/m^3^)	SHV (R)	UCS (MPa)			PLI (MPa)			ITS (MPa)		
					N	D	S	N	D	S	N	D	S
MLS	Min.	2.3	2.3	48	24.5	62.4	23.1	3.6	3.8	3.3	4.3	6.5	2.9
	Max.	2.6	2.6	66	58.3	153.7	50.1	7.5	6.6	7.7	10.7	12.5	10.9
	Avg.	2.5	2.6	59	40.1	101.2	32.8	5.7	5.6	5.1	8.6	8.2	6.7
FLS	Min.	2.4	2.4	49	57.4	28.7	32.6	3.8	4.6	1.8	6.3	5.6	1.6
	Max.	2.7	2.7	67	178.9	93.8	44.8	7.4	7.0	8.3	12.8	11.0	10.7
	Avg.	2.6	2.6	61	95.3	47.9	36.8	6.1	6.1	6.0	10.1	8.7	6.8
SLS	Min.	2.2	2.2	50	17.7	49.2	19.7	1.1	5.0	1.6	4.7	5.7	2.1
	Max.	2.7	2.7	76	112.2	220.9	87.1	11.3	14.6	12.1	13.0	17.2	14.8
	Avg.	2.4	2.4	62	39.3	119.9	36.4	5.9	7.0	6.1	9.2	9.8	7.7

N = natural; D = dry; S = saturated.

**Fig. 3:**
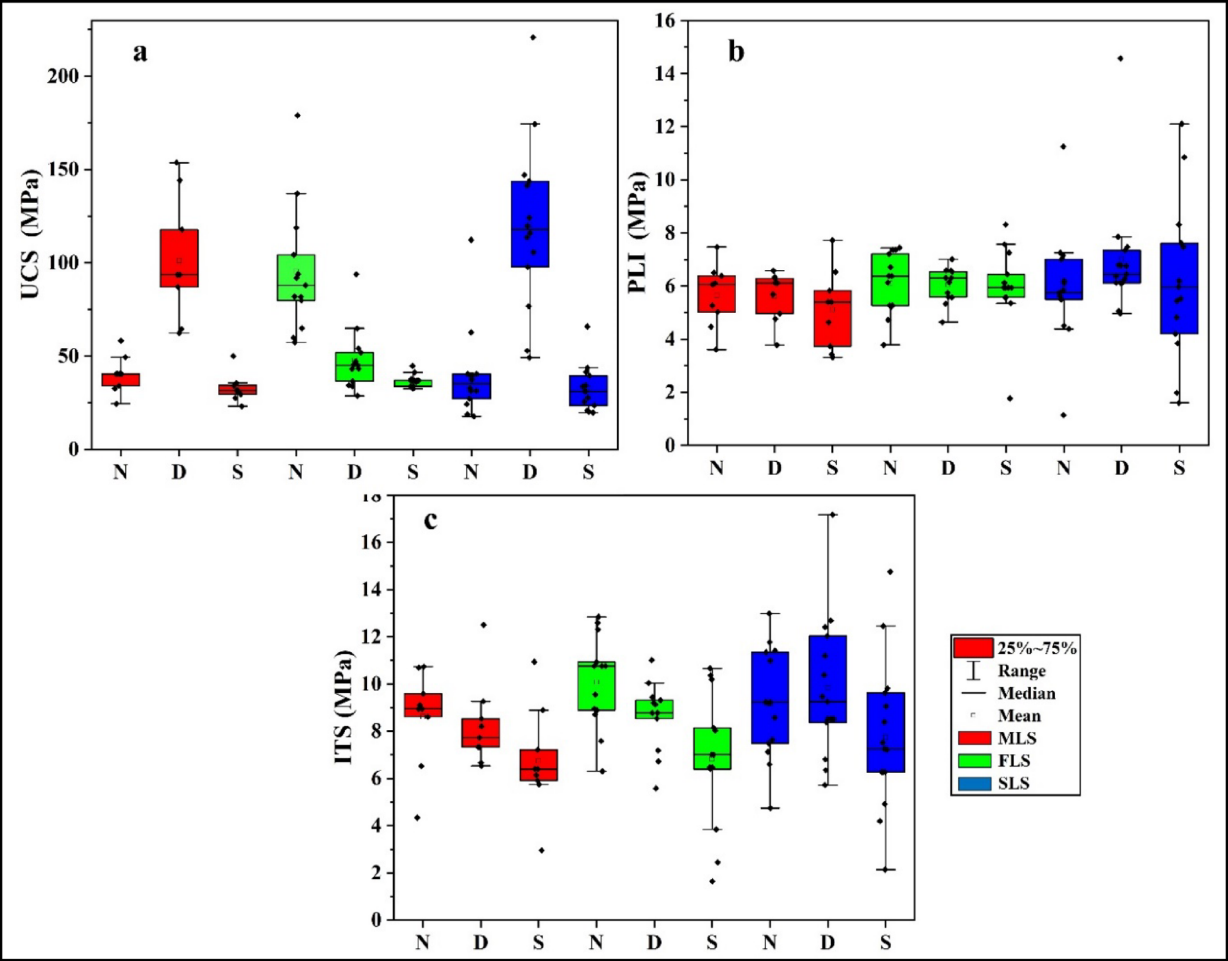
Mechanical properties of the MLS, FLS, and SLS lithofacies. (**a**) UCS; (**b**) PLI; and (**c**) ITS.

Figure 3 shows the variations in UCS, PLI, and ITS for the three lithofacies under natural (N), dry (D), and saturated (S) conditions. The highest UCS, PLI, and ITS values occurred when dry conditions eliminated water-induced weakening in all lithofacies. Dry conditions slightly enhanced the PLI compared with natural conditions. Conversely, the UCS and ITS values decreased when the samples reached saturation. The PLI is a localised test; thus, it was less affected by saturation.

The differences between natural and dry conditions were most pronounced in MLS, where UCS under dry conditions was almost three times higher than under natural conditions. The UCS values under natural and saturated conditions were relatively similar. This can be attributed to the low porosity of MLS, which implies a low storage capacity for water. Oven drying at 105 °C eliminates the residual water from microfractures and grain boundaries, increasing grain interlocking and leading to the observed large increase in UCS. In contrast, PLI values changed only slightly, because they are controlled by localized point loading, which is less sensitive to the bulk porosity or water content. ITS in MLS remained lower than in other lithofacies because tensile strength is strongly fracture-controlled, and the orientation and connectivity of pre-existing cracks override the porosity and water content effects. By comparison, FLS, with a higher fossil-related porosity, displayed a larger difference between natural and saturated strengths, highlighting the role of pore structure on water sensitivity^47^.

The mechanical parameters of FLS varied widely, thereby indicating higher variability owing to fossil heterogeneity. Saturation may weaken calcite-cemented contacts, resulting in a substantial strength reduction. The UCS of FLS values under dry conditions were systematically lower than those of MLS and SLS. Petrographic and thin-section observations revealed the presence of intra-fossil pores and weak fossil–matrix interfaces, which could serve as preferential failure planes during loading. Fossil content was not measured for each specimen, but the observation of abundant bioclasts and intra-fossil pores in all FLS thin-sections demonstrates that this lithofacies is by nature more porous and more mechanically heterogeneous than MLS and SLS. These textural features explain both the lower dry UCS and the wider variability of UCS and ITS values observed in FLS. The presence of tensile failure planes at fossil interfaces resulted in the lowest ITS values among the lithofacies. The higher ITS values obtained under natural conditions relative to those under dry conditions can be attributed to residual moisture, which may enhance ductility and delay crack development. Water saturation impairs this effect by causing debonding between the fossil and matrix, which enhances anisotropic behaviour.

SLS had the highest overall strength under dry conditions owing to its rigid siliceous structure. The interaction of water with silica caused only minor changes, as silica exhibits low reactivity to water. Therefore, the reduction in UCS of SLS under saturated conditions cannot be attributed to silica–water interaction. The weakening is related to the calcite matrix that bonds siliceous grains, and to the minor clay and iron oxide impurities that were revealed by XRF analyses. These impurities can soften when wet and weaken grain-boundary strength. Furthermore, water can infiltrate microcracks and thus promote stress concentration and crack propagation. Although silica itself is inert, the carbonate matrix and minor impurities present in SLS are accountable for the UCS reduction under saturated conditions. The ITS remained stable, thereby reflecting a lack of vulnerability to crack propagation. The durability of the SLS facies was enhanced through siliceous cementation, which made it the most resilient lithofacies under all conditions analysed herein.

Saturation produced lower UCS and ITS values, as water infiltration diminishes cohesion and advances crack development^14,48,49^. MLS underwent substantial weakening under saturation, primarily owing to fracture penetration, whereas FLS was the most vulnerable due to high porosity from its fossil components. SLS outperformed the other lithofacies under all conditions because the inherent rigidity of its siliceous structure minimised water-induced degradation. It is noteworthy that the difference between ITS values under dry and saturated conditions was smaller than that observed for UCS. This apparent inconsistency reflects the different failure mechanisms: UCS is strongly reduced by water because cohesion and grain interlocking are weakened throughout the rock mass. In contrast, ITS performance shows no significant variation with water content, since it remains largely controlled by crack initiation and propagation along pre-existing flaws. Thus, while saturation accelerates UCS reduction, its effect on ITS is relatively minor, since tensile failure is already controlled by structural weaknesses such as fossil–matrix boundaries, fractures, or grain contacts.

Owing to its highly variable strength caused by its fossil content, FLS is the least reliable material for load-bearing applications. MLS would require stabilisation in environments with water exposure, whereas SLS is best suited for structures that may be exposed to moisture, such as dams and foundations. Water saturation universally weakens rock strength; however, the degree of weakening depends on the facies properties. In this study, FLS exhibited the largest decrease in strength, followed by MLS (moderate decrease) and SLS (minimal decrease). The oven-drying process at 105 °C removed moisture and improved grain interlocking, particularly in SLS, since silica maintains its stability at high temperatures. These observations are in accordance with those of previous studies, thereby reinforcing the finding that moisture plays a critical role in limestone strength and deformation behaviour^28–30,50^. Vásárhelyi and Ván^51^ found that water can reduce the UCS of porous rocks by 10–60% as a result of pore pressure effects combined with chemical weakening and crack propagation. Abd El Aal et al.^29^ recorded that UCS decreased (up to 36%) between the dry and saturated stages of the Jurassic limestones of Saudi Arabia. The strength decreases observed in all three lithofacies under saturated conditions support this finding. Rabat et al.^49^ demonstrated that water saturation produces substantial decreases (30–56%) in both direct and indirect tensile strengths in porous limestones. Tensile weakening due to water exposure varied among the lithofacies in this study owing to their distinct petrological and physical properties. The observed strength variability across facies results from specific factors, including pore structures, fossil–matrix bonding, and geochemical composition.

### Statistical analysis

Previous studies have demonstrated the relevance of bulk geochemistry in predicting rock strength, particularly in carbonates where compositional purity, cementation, and impurity content play significant roles^52,53^. Statistical and empirical models, including regression analyses and Taylor diagrams, have been used to successfully predict mechanical properties (such as UCS, Young’s modulus, and tensile strength) from bulk geochemical and physical data in carbonate formations^52^. Hussain et al.^53^ proposed a workflow that statistically integrates geochemical and mechanical datasets to predict rock strength from geochemical data. This approach is described as simple, cost-effective, and time-efficient, allowing for the rapid establishment of a proxy for rock strength using geochemical and mechanical properties of rocks, including carbonates. In this study, the mechanical properties of the lithofacies (i.e. UCS, PLI, and ITS) were affected by their chemical compositions under natural, dry, and saturated conditions. Pearson correlation analysis was used to clarify the relationships between the major oxide contents and the mechanical properties of the three lithofacies (Table 3). The presence of MgO increased the PLI values across all conditions, as well as ITS under natural conditions, which demonstrates the contribution of dolomitization to improving limestone rock strength. Under natural conditions, CaO was moderately positively correlated with UCS (*r* = 0.342), but was negatively correlated with UCS (*r*=-0.462) and PLI (*r*=-0.216) under dry conditions, which is evident in the decreases in the UCS and PLI values of FLS under dry conditions. SiO_2_ was negatively correlated with UCS (*r*=-0.331) under natural conditions. Cl was strongly correlated with PLI under dry conditions, thereby indicating its strengthening capacity in dry environments. The mechanical properties of the lithofacies exhibited weak or context-dependent correlations with constituents such as Al_2_O_3_, Fe_2_O_3_, Na_2_O, P_2_O_5_, SO_3_, and insoluble residues.

**Table 3:** Pearson correlation coefficients obtained between UCS, PLI, and ITS and the chemical compositions of the limestone lithofacies.

	UCS (MPa)			PLI (MPa)			ITS (MPa)		
	*N*	D	S	*N*	D	S	*N*	D	S
CaO	**0.342** ^*^	**– 0.462** ^**^	– 0.054	– 0.069	– 0.216	– 0.173	– 0.007	– 0.020	– 0.111
SiO_2_	**– 0.331** ^*^	**0.493** ^**^	– 0.137	– 0.104	– 0.006	0.112	– 0.074	– 0.002	0.100
MgO	-0.131	0.100	**0.679** ^**^	**0.561** ^**^	**0.833** ^**^	**0.362** ^*^	0.279	0.075	0.134
Al_**2**_**O**_**3**_	– 0.258	0.239	– 0.279	– 0.129	– 0.122	– 0.146	– 0.121	0.019	-0.044
Fe_**2**_**O**_**3**_	– 0.276	**0.328** ^*^	– 0.252	– 0.086	– 0.097	– 0.067	– 0.161	0.048	– 0.041
Na_**2**_**O**	0.090	-0.067	0.102	0.044	– 0.173	0.071	0.117	– 0.237	0.106
P_**2**_**O**_**5**_	– 0.295	0.152	– 0.131	-0.118	0.115	– 0.152	0.058	– 0.006	– 0.043
SO_**3**_	0.162	0.268	– 0.067	0.050	– 0.116	0.072	0.122	0.061	0.150
Cl	– 0.233	0.216	0.280	**0.354** ^*^	**0.556** ^**^	0.140	0.071	0.253	0.182
IR	– 0.130	0.288	– 0.074	– 0.009	-0.057	0.061	0.039	– 0.109	– 0.013

N = natural, D = dry, S = saturated.

Signficant values are in bold.

**Fig. 4:**
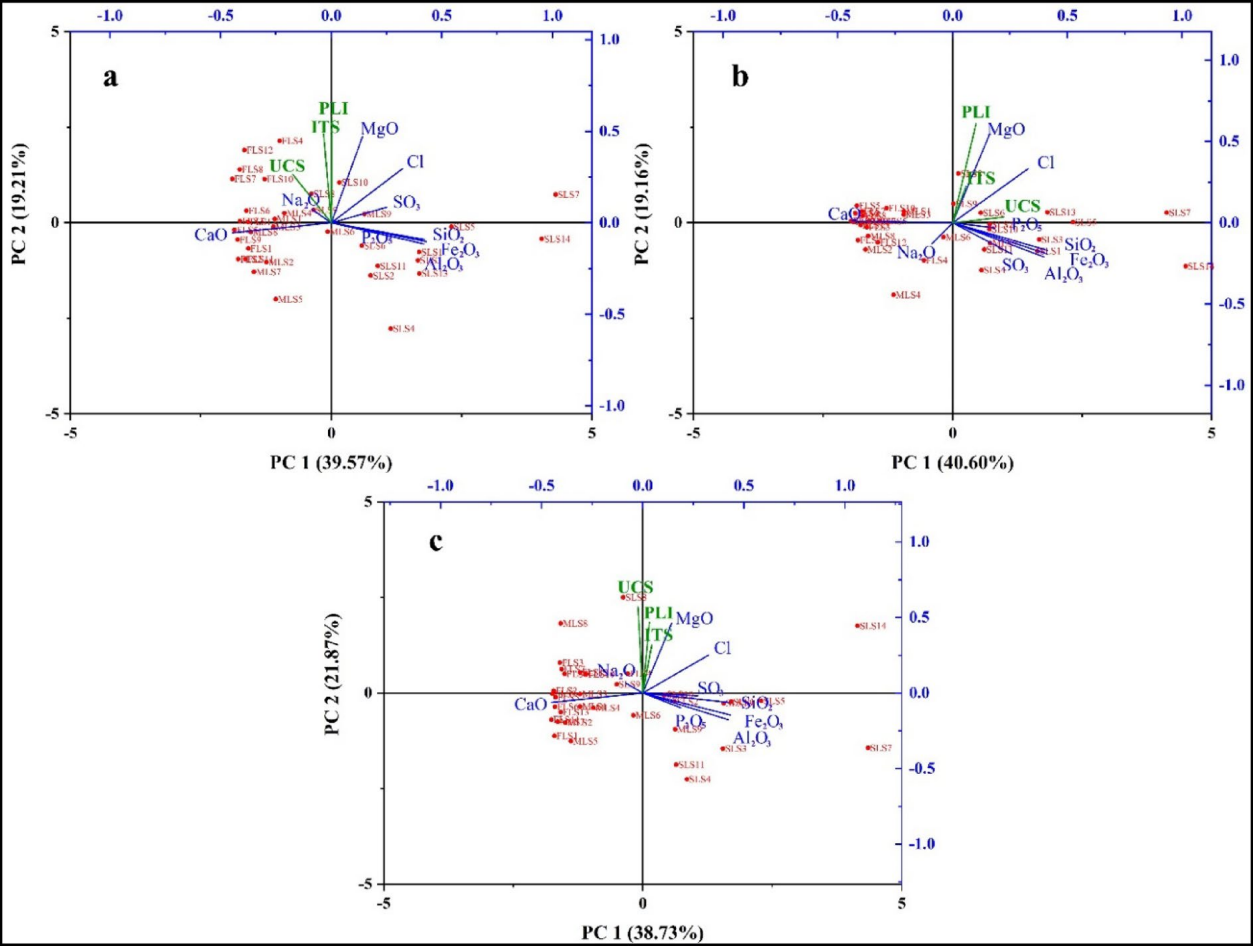
Principal Component Analysis (PCA) plots showing the relationships between the rock mechanical properties (UCS, PLI, and ITS) and chemical compositions of the lithofacies under (**a**) natural, (**b**) dry, and (**c**) saturated conditions. The red color represents the sample numbers.

Table 4; Fig. 4 show the multivariate Principal Component Analysis (PCA) between the mechanical properties (UCS, PLI, and ITS) and chemical compositions (major oxides) of the limestone lithofacies under natural, dry, and saturated conditions. In the PCA, the first two principal components (PC1 and PC2) together explain 58.78%, 59.76%, and 60.60% of the total variance for natural, dry, and saturated conditions, respectively. PC1 is primarily defined by positive loadings of SiO_2_, Al_2_O_3_, and Fe_2_O_3_ and negative loadings of CaO, indicating a contrast between carbonate purity and limestone impurities. PC2 is dominated by secondary contributions from MgO, reflecting a minor composition that influences strength.

**Table 4:** Loadings, eigenvalues, and variance, percentage for PCA.

	Natural		Dry		Saturated	
	PC1	PC2	PC1	PC2	PC1	PC2
Eigenvalue	4.749	2.306	4.872	2.300	4.647	2.624
Variance (%)	39.57	19.21	40.60	19.16	38.73	21.87
CaO	**– 0.4463**	– 0.0540	**– 0.4419**	0.0126	**– 0.4516**	– 0.0618
SiO2	**0.4223**	– 0.0923	**0.4137**	– 0.1695	**0.4272**	– 0.0623
MgO	0.1416	**0.4722**	0.1618	**0.5486**	0.1436	**0.4644**
Al_2_O_3_	**0.4189**	– 0.1160	**0.3978**	– 0.2103	**0.4230**	– 0.1762
Fe_2_O_3_	**0.4286**	– 0.1028	**0.4115**	– 0.1917	**0.4328**	– 0.1454
Na_2_O_3_	– 0.0887	0.0739	– 0.0908	– 0.1296	– 0.0848	0.0663
P_2_O_5_	0.1938	-0.0432	0.1837	– 0.0306	0.1881	– 0.0970
SO3	0.2504	0.0850	0.2577	– 0.1920	0.2718	– 0.0183
Cl	0.3220	0.2960	0.3292	0.3335	0.3264	0.2516
UCS	– 0.1738	0.2635	0.2220	0.0352	– 0.0236	**0.5707**
PLI	0.0024	**0.5778**	0.1017	**0.6093**	0.0329	**0.4696**
ITS	-0.0342	**0.4860**	0.0583	0.2257	0.0449	0.3175

Signficant values are in bold.

UCS, PLI, and ITS clustered around specific oxides, indicating that they have variable relationships under different conditions. SLS was strongly aligned with SiO_2_, Fe_2_O_3_, and Al_2_O_3_; thus, its strength varies with impurities across different conditions. The mechanical properties of FLS and MLS exhibited differing levels of response to CaO and MgO, thereby indicating that the limestone’s purity affects its mechanical properties. The PCA plots indicate that Fe_2_O_3_ and Al_2_O_3_ have minor influences on strength, which implies that they are not essential to mechanical performance. Under natural conditions, UCS, PLI, and ITS were aligned with CaO and MgO, particularly in MLS and FLS. The strong alignment between SLS and SiO_2_ indicates that the silica content plays a major role in rock strength. The effect of SiO_2_ decreases under dry conditions since quartz grains react less to drying than carbonates. Water saturation adds complications, which increases the importance of pore structures and mineral reactivity compared to that of the oxide content.

The statistical results support geological interpretations in which the chemical composition (including carbonate and silica contents) affects the mechanical properties of limestone lithofacies. The strength characteristics exhibited different responses under natural, dry, and saturated conditions because the lithofacies mineralogy, combined with moisture sensitivity, has a large effect on the mechanical properties. Engineers use this knowledge to assess tunnel stability and design foundations in carbonate terrains, as well as to analyse slope stability and evaluate rock mass integrity at dam abutments and underground excavations. Geomechanical models have become more reliable as the relationships between geological and geotechnical contexts have been clarified.

### Practical applications

#### Geological studies

##### Lithofacies characterisation

The observed differences in mechanical and geochemical properties among the MLS, FLS, and SLS samples demonstrate why lithofacies classification is vital for geological surveying. Understanding these variations enables scientists to reconstruct depositional environments and diagenetic histories, which are essential for effective hydrocarbon exploration and reservoir characterisation.

##### Weathering and durability

According to the findings of this study, environmental conditions, including water saturation, play a critical role in modifying rock strength. Understanding these relationships is vital for predicting long-term weathering patterns and evaluating the stability of carbonate rocks across various climatic regions.

#### Engineering projects

##### Infrastructure design

Structural materials exposed to moisture are required to have exceptional performance characteristics. As determined herein, SLS exhibited superior strength and durability under saturated conditions; thus, is recommended for critical applications such as dams, bridges, and foundations. FLS would require stabilisation or alternative materials, as its high porosity makes it susceptible to water-induced weakening in such environments.

##### Slope stability and tunnelling

Slope stability evaluations and tunnel construction design benefit from studying the variations in UCS, PLI, and ITS between different lithofacies. Under saturation, the fracture-based strength of MLS decreased; therefore, MLS would require reinforcement during excavation.

##### Predictive modelling

The relationships between chemical components (e.g. SiO_2_ and MgO) and rock mechanical properties can be used to establish precise geomechanical models. Such models could then be used to simulate rock behaviour under various loading and environmental conditions to improve risk management strategies.

#### Relevant industries

##### Within the fields of construction and mining

This study established a selection methodology for identifying suitable limestone lithofacies for use as construction aggregates, dimensional stones, and other industrial components. SLS demonstrates superior resilience for high-stress applications, whereas FLS is preferable for decorative uses where strength requirements are minimal.

##### In the oil and gas industry

Drilling efficiency and reservoir integrity depend on the mechanical properties of the carbonate host rock. Understanding how saturation affects these mechanical properties allows for better predictions of wellbore stability and more effective hydraulic fracturing designs for carbonate reservoirs.

##### In heritage conservation

Understanding carbonate weathering mechanisms helps preserve historical structures and monuments, particularly in regions with extreme moisture variations.

### Limitations and future research

This study had several limitations that require further investigation. First, a representative sample size was used herein, but sampling was constrained to one geographic location (Hafit Mountain). Additional investigations would help determine the wider relevance of the findings to global limestone lithofacies variability. Future studies should include samples from multiple areas to validate these findings. In addition, short-term saturation effects were investigated in this study, but long-term experiments simulating natural weathering processes (e.g. freeze–thaw cycles and chemical dissolution) could yield a better understanding of limestone durability. Another limitation is that fossil abundance was not quantified on a per-specimen basis. The lithofacies classification was based on uniform petrographic evidence of abundant intra-fossil pores and weak fossil–matrix interfaces in FLS. Although this method is appropriate to capture the representative mechanical behaviour of the lithofacies, it is not sufficient to record specimen-specific fossil content data. Future work should integrate quantitative fossil abundance measurements (i.e. automated image analysis of thin-sections, or micro-CT porosity mapping) to more accurately assess the impact of fossil proportion and spatial distribution on mechanical variability in fossiliferous limestones. Finally, the microstructural attributes of the lithofacies, including pore structure and cementation patterns, were not addressed in detail in this study but should be examined in future studies. The application of advanced imaging methods, such as micro-CT scanning, has the potential to clarify any connections between these factors. Predictive models utilising machine learning techniques should also be developed to improve the strength prediction accuracy across different environmental conditions.

## Conclusions

The findings of this study indicate that lithofacies type and environmental conditions strongly affect the mechanical properties of carbonate rocks. The silica content of SLS allowed it to maintain its superior strength under all conditions analysed herein; thus, it is the best option for construction materials that are exposed to moisture. FLS exhibited poor mechanical performance owing to its high porosity and fossil content, which intensified under saturated conditions. MLS exhibited decreases in strength that were dependent on fracture development. Water saturation decreased rock strength, the extent of which was dependent on the lithofacies characteristics. Dry conditions enhanced rock strength by improving grain interlocking, particularly in SLS. The mechanical properties of the limestone samples varied based on their geochemical compositions (e.g. CaO, SiO_2_, and MgO); therefore, mineralogical and chemical indicators can be used effectively for predictive modelling. SiO_2_ and MgO were major mechanical behaviour indicators, as silica enhances rock durability and dolomitization improves tensile strength. A robust framework for evaluating carbonate rocks for engineering applications was established. The findings obtained herein enable the identification of appropriate lithofacies for construction, mining, and hydrocarbon extraction, which increases the safety and sustainability of engineering projects. Engineers must therefore consider both lithofacies variability and environmental exposure conditions when selecting carbonate materials for construction projects. This study advances the field through developing a mechanistic framework linking lithofacies composition, microstructural attributes, and strength response under different moisture conditions. This framework extends beyond the studied site and provides a conceptual foundation for predictive modeling carbonate durability in engineering and environmental contexts.

## Data Availability

The datasets generated and/or analyzed during the current study are available from the corresponding author on reasonable request.
